# Density–Density Correlation Spectra of Ultracold Bosonic Gas Released from a Deep 1D Optical Lattice

**DOI:** 10.3390/e26100854

**Published:** 2024-10-10

**Authors:** Yunzhi Tan, Qiang Zhu, Bing Wang, Jingran Shi, Dezhi Xiong, Baolong Lyu

**Affiliations:** 1 Key Laboratory of Atomic Frequency Standards, Innovation Academy for Precision Measurement Science and Technology, Chinese Academy of Sciences, Wuhan 430071, China; tanyunzhi19@mails.ucas.ac.cn (Y.T.); zhuqiang@apm.ac.cn (Q.Z.); wangbing@apm.ac.cn (B.W.); shijingran@apm.ac.cn (J.S.); baolonglyu@apm.ac.cn (B.L.); 2 University of Chinese Academy of Sciences, Beijing 100049, China; 3 Hefei National Laboratory, Hefei 230088, China

**Keywords:** density–density correlation, Mott insulator, 1D optical lattices

## Abstract

Density–density correlation analysis is a convenient diagnostic tool to reveal the hidden order in the strongly correlated phases of ultracold atoms. We report on a study of the density–density correlations of ultracold bosonic atoms which were initially prepared in a Mott insulator (MI) state in one-dimensional optical lattices. For the atomic gases released from the deep optical lattice, we extracted the normalized density–density correlation function from the atomic density distributions of freely expanded atomic clouds. Periodic bunching peaks were observed in the density–density correlation spectra, as in the case of higher-dimensional lattices. Treating the bosonic gas within each lattice well as a subcondensate without quantum tunneling, we simulated the post-expansion density distribution along the direction of the 1D lattice, and the calculated density–density correlation spectra agreed with our experimental observations.

## 1. Introduction

For easier detection, ultracold atomic gases confined in external trapping potentials are often released into free space before being probed. This is usually performed by suddenly switching off the trap to allow the atomic cloud to expand freely for a certain time of flight (TOF). A probe laser is then applied to take an absorption image which records the two-dimensional (2D) column density of the expanded cloud. Interference patterns in the atomic density distribution can be used to identify the single particle coherence of the in-trap gases. However, for many-body phenomena beyond single particle coherence, the density profile of an expanding cloud usually shows no interference pattern, providing little useful information on the properties of the involved quantum states. Instead, the spatial correlation function of the density fluctuations, i.e., the density–density correlation function, was proposed as a means to probe the hidden order of the complex many-body states of trapped ultracold atoms [1]. In experiments, density–density correlation analysis has been used to monitor the establishment of the Mott insulator (MI) phase of ultracold atoms in three-dimensional (3D) lattices [2] and 2D lattices [3], and an array of sharp peaks at the positions of the integer multiples of the reciprocal lattice vector were observed in the density–density correlation spectra when the lattice system reached the MI phase. At single-atom detection level, a periodic high-contrasted bunching is observed to occur in two-body and three-body correlations for 3D He*-4 Mott states [4]. Recently, pairs in the depletion of an interacting Bose gas [5] and strongly correlated Fermi cooper pairs [6] were revealed by measuring density–density correlations.

The technique of density–density correlation has also been applied to in-trap gases. The in–trap density distribution can be obtained from an in situ image of a trapped gas without free expansion, but a more sophisticated imaging system is usually required. Many novel quantum phenomena have been found using density–density correlation analysis of a variety of quantum systems. For instance, peculiar long-range patterns of density–density correlation function have been predicted as evidence of Hawking radiation from sonic black holes in a flowing one-dimensional (1D) atomic Bose–Einstein condensate (BEC) [7]. Recently, both self-amplifying and spontaneous Hawking radiations of phonons were observed from such analogue black holes by measuring density–density correlation patterns [8,9].

It is well known that a retroreflected laser beam forms the simplest 1D optical lattice, which consists of a chain of disk-shaped traps. Such an optical lattice confining ultracold bosonic atoms in a single spin state is one of the typical lattice systems that can be described by the Bose–Hubbard model. In contrast to a higher-dimensional optical lattice, the MI phase of a 1D lattice system is harder to achieve, due to both the much weaker on-site interaction and the much larger atomic number at each lattice site [10,11]. For condensates confined in a deep 1D optical lattice, blurred or vanished interference patterns were witnessed when the bosonic gases were released from the optical lattice [12]. The vanishing of the contrast of the interference patterns was once considered as the characteristic of MI states [12,13]. However, Gerbier et al. found, in their experimental work [13], that a finite visibility could still be observed even deep in the MI phase, due to the existence of particle–hole pairs. They then reproduced their observations in theory using a random phase approximation [14]. In contrast to single particle coherence, density–density correlation seems to be a better means for gaining a deeper insight into the MI phase. The density–density correlation function of the bosonic gases released from a 1D optical lattice was calculated under the assumption that the system was initially prepared in a Fock state [15]. In particular, for the case of a MI phase with an equal number of atoms at each lattice site, the predicted correlation function consists of regular sharp peaks, analogous to the interference pattern of an optical grating. To our knowledge, however, no experimental works have been reported on the density–density correlation spectra of 1D optical lattice systems.

In this work, we report a detailed study on the density–density correlation of a pure ^87^Rb condensate adiabatically loaded into a deep 1D optical lattice. For the freely expanded gases released from the lattice, periodic bunching peaks were observed along the lattice direction, as expected for the MI phase. The amplitude of correlation peaks was significantly larger than that of 3D [2] or 2D [3] lattice systems. We also developed a theoretical model to simulate the atomic density distribution. The density–density correlation spectra calculated from the simulated density profiles agreed with the observed spectra.

## 2. Experiments

The experiment sequence started with loading a pure condensate of ^87^Rb atoms into a 1D optical lattice. The condensate typically contained $N=1.3\times 10^{5}$ atoms of $|F=2,m_{F}=2\rangle$ state. It was created in a magnetic trap with axial and radial trapping frequencies of 2π×{18.7,205} Hz. To increase the size of the condensate and hence allow the condensate to occupy more lattice sites, we subsequently adiabatically relaxed the magnetic trap to lower trap frequencies of $\left\{\omega_{x},\omega_{r}\right\}=2\pi \times \left\{8.2,90.4\right\}$ Hz. Thus, a cigar-shaped BEC was formed with axial and radial Thomas–Fermi diameters of 96 μm and 9 μm, respectively. The 1D optical lattice was formed by a retroreflected λ=1064 nm laser beam with a waist radius of 140 μm. The lattice depth $V_{0}$ was calibrated using the conventional method of Kapitza–Dirac scattering [12], and written in units of the recoil energy ($V_{0}=sE_{r}$), where the recoil energy $E_{r}=h^{2}/\left(2m\lambda^{2}\right)$, and *m* is the atomic mass. To load the condensate into the 1D optical lattice, we applied the optical lattice along the axis (*x* direction) of the relaxed condensate by linearly ramping up the lattice depth to a given value over a time of 30 ms and holding it fixed for 10 ms. The adiabatic condition of the loading process was $dV_{0}/dt_{0}\ll 16E_{r}^{2}/\hbar$ [16], where $V_{0}$ is the final trap depth and $t_{0}$ is the ramping time. For our ramping-up time, $dV_{0}/dt_{0}$ was about 50 times smaller than $16E_{r}^{2}/\hbar$, which satisfied the adiabatic condition.

For such an ultracold bosonic gas loaded in a 1D optical lattice, the mean occupation $\bar{n}$ is much larger than one. The quantum gas in a single lattice well can be treated as a subcondensate, and the whole system can be considered as a chain of subcondensates. The critical point of the Mott phase transition from the superfluid phase was studied in early theoretical works, and is given as follows [11,14]:

(1) $$U/J=4\bar{n}+2+4\sqrt{\bar{n}\left(\bar{n}+1\right)}.$$

Here, the on-site interaction energy *U* is written as [17,18]

$$U=\frac{1}{3}{\left(\frac{2}{\pi }\right)}^{3/4}\sqrt{\frac{gm\omega_{⊥}^{2}}{\sigma_{x}\bar{n}}},$$

where $g=4\pi \hbar^{2}a/m$ is the interaction parameter, *a* is the s-wave scattering length, and $\sigma_{x}=\lambda /\left(2\pi s^{1/4}\right)$ is the axial Gaussian width of the wave packet at a lattice site. The tunneling energy *J* can be approximated by [17,19]

$$J=\frac{4}{\sqrt{\pi }}E_{r}s^{3/4}e^{-2\sqrt{s}}.$$

Using the experimental parameter $\bar{n}\approx 530$, the critical value ${\left(U/J\right)}_{c}\approx 4.2\times 10^{3}$, and the corresponding lattice depth was about s=96. If U/J was well below ${\left(U/J\right)}_{c}$, the phases of the subcondensates in single wells were correlated, giving a momentum distribution with interference patterns of sharp peaks [12]. For $U/J\gg {\left(U/J\right)}_{c}$, the subcondensates were well localized, and the phase of the subcondensate in each well was completely random. Our final lattice depth was s=132, corresponding to $U/J\approx 1.2\times 10^{5}$. Thus, atomic tunneling between adjacent lattice wells was highly suppressed ($J/h\approx 1.9\times 10^{-5}$ Hz), and the lattice system was deep in the MI regime.

**Figure 1:**
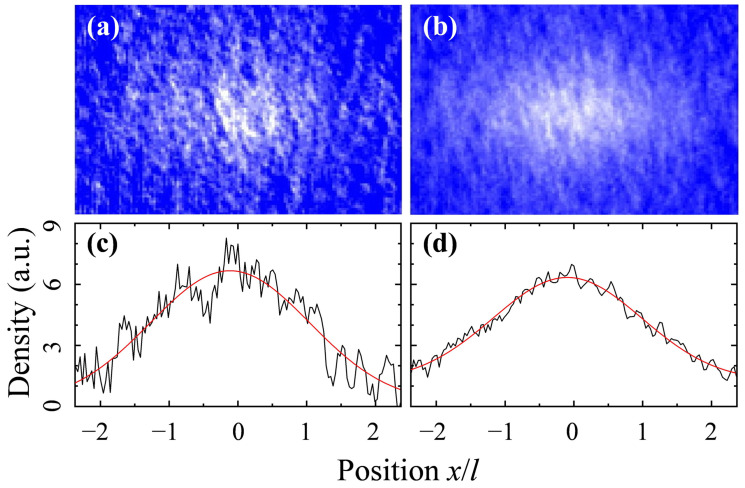
(**a**) Single shot absorption image showing density distribution of ^87^Rb atoms. (**b**) The averaged density distribution of 30 images taken repeatedly under the same experimental conditions. (**c**,**d**) are atomic density profiles in the horizontal direction, which were obtained by integrating the column density in (**a**,**b**) along the vertical direction, respectively. The red lines in (**c**,**d**) are the Gaussian fits to the corresponding atomic density profiles.

We then suddenly switched off the combined potential, and allowed the atomic gas to expand for τ=30 ms. Then, the expanded atomic cloud was probed using the conventional absorption imaging technique, as shown in Figure 1a. The probe light was applied along the *z*–direction, and the atomic density was recorded using a CCD camera. In fact, the atomic density distribution reflects the momentum distribution of the in-trap gas. Figure 1b displays the averaged atomic density distribution, which showed less density fluctuations in contrast to the single-shot image.

Spatial density–density correlation is related to the probability of finding one particle at a certain location given that another particle is present at some other locations. We extracted the normalized density–density correlation function by analyzing a set of images taken in repeated experiments. We first integrated the column density of a single shot image along the vertical direction to obtain an atomic density profile in the horizontal direction (see Figure 1c). Then, the normalized density–density correlation function was calculated from the obtained density profiles according to the following formula [2]

(2) $$C\left(\Delta x\right)=\frac{\int \langle n(x)n(\Delta x+x)\rangle \;dx}{\int \langle n(x)\rangle \langle n(\Delta x+x)\rangle \;dx},$$

where n(x) is the atomic density at the position *x* in a single density profile, Δx stands for the separation between two positions, and 〈〉 donates a statistical averaging over a set of independent profiles. A Butterworth high-pass filter was applied to the correlation signal to eliminate the Gaussian background.

**Figure 2:**
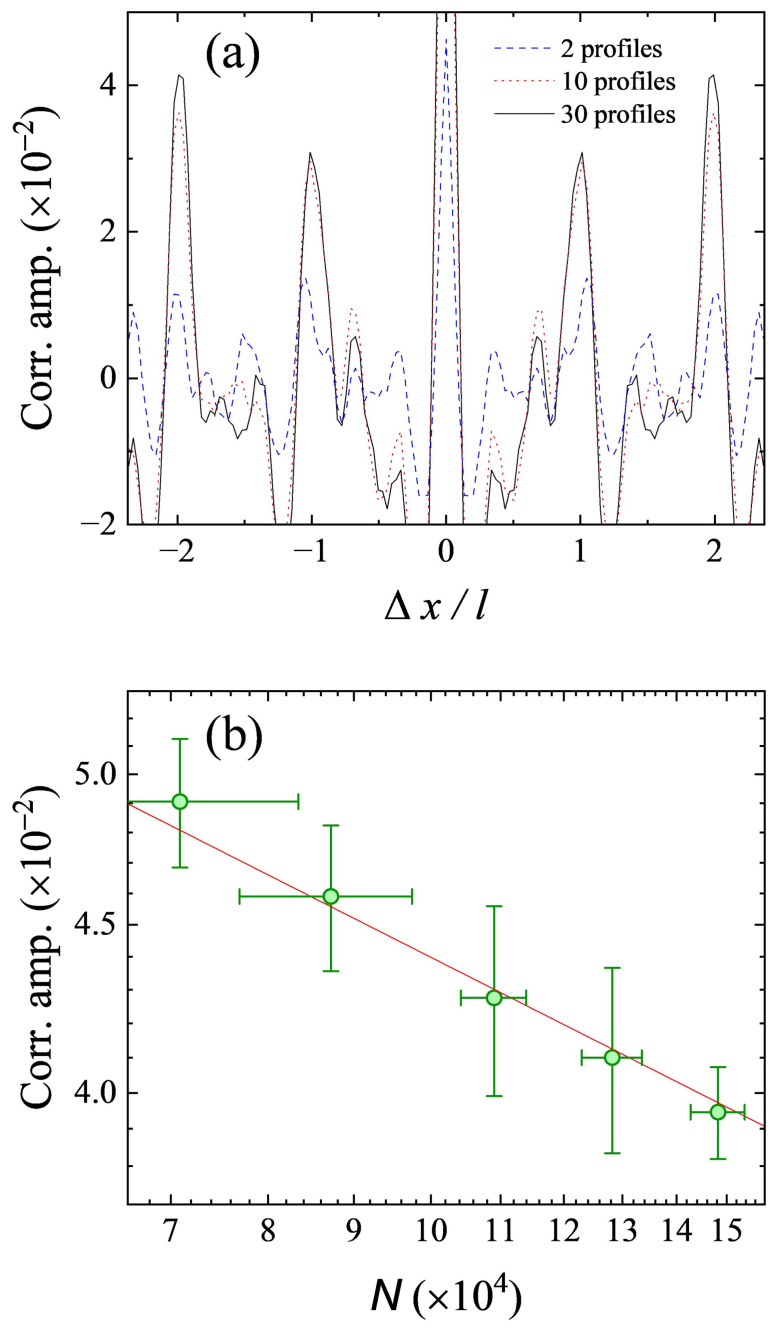
(**a**) Density–density correlation spectra extracted from 2, 10, and 30 independent density profiles, respectively. Each density profile is similar to the one in Figure 1c. (**b**) The amplitude of density–density correlation peaks versus atom number *N*. The error bars correspond to one standard deviation. The red line is a fitted power–law decaying curve in the form of $N^{-\alpha }$.

The numerator of Equation (2) was determined by calculating the autocorrelation function (ACF) of each 1D density profile separately, and then averaging over many realizations, while the denominator was determined by calculating the ACF of the average of these 1D density profiles (as shown in Figure 1d). Figure 2a shows the obtained density–density correlation function C(Δx) of the rubidium gases. As anticipated, the correlation peaks in the density–density correlation emerged at the position of the integer multiples of the reciprocal lattice vector $k_{L}=2\pi /d$, with d=λ/2 being the lattice period. In addition, the reciprocal lattice vector sets the characteristic length $l=\hbar k_{L}\tau /m$, which is the spatial separation of diffraction peaks when the system is coherent. The periodic peaks arise from the bunching effect of bosonic atoms, revealing the spatial order of the in-trap atoms.

In order to find out how many absorption images were necessary to obtain a reliable correlation signal, we checked the dependence of the correlation signal on the number of absorption images used. The amplitude of correlation peaks increased with an increasing image number at first, but soon became basically unchanged when the image number reached 10. The peak height was about 0.04 (see the black solid line in Figure 2a), which is about two orders larger than that of 2D and 3D lattice systems [2,20]. This is because the correlation signal amplitude is proportional to $1/M^{D}$ [21], where *D* is the dimension of the lattice, and *M* is the number of lattice sites occupied by the condensate in one dimension.

The resolution of our imaging system was mainly determined by the pixel size of the CCD camera ($\sigma_{\mathrm{px}}=9\;\mu$m), whereas the predicted peak width of the density–density correlation signal for a 1D lattice system is only l/M≈1μm. If the imaging resolution is taken into consideration, the amplitude of correlation peaks can be approximated by $l/(2M\sigma_{\mathrm{px}}$) [21], which yields a value of ∼0.06, and is roughly in agreement with our experimental value (∼0.04).

The amplitude of correlation peaks is also affected by the total number of atoms *N* loaded in the 1D lattice. A detailed theoretical analysis will be presented in next section. According our theoretical model, the peak height $C(\Delta x_{\mathrm{peak}})$ should scale as $N^{-\alpha }$. We experimentally measured the peak heights for different values of *N* (see Figure 2b). The power-law fitting to the data points yielded an exponent of 0.26±0.06, close to the theoretical value (α=0.2).

## 3. Theory and Simulation

We here try to use numerical simulation to reproduce the normalized density–density correlation spectra. With a zero-tunneling limit, the 1D atomic lattice system can be understood as a chain of disk-shaped subcondensates with completely random phases. The subcondensates are spaced by the lattice period d=λ/2, and occupy $M=2k_{M+1}$ lattice sites ($-k_{M}$ to $k_{M}$). The atom number in the *k*th lattice site is given by [17,22]

$$N_{k}=\frac{15N}{16k_{M}}{\left(1-\frac{k^{2}}{k_{M}^{2}}\right)}^{2},$$

while $k_{M}$ takes the following form

(3) $$k_{M}^{2}=\frac{2\hbar \bar{\omega }}{m\omega_{x}^{2}d^{2}}{\left(\frac{15N}{8\sqrt{\pi }}\frac{a}{a_{\mathrm{ho}}}\frac{d}{\sigma_{x}}\right)}^{2/5},$$

where $\bar{\omega }={\left(\omega_{x}\omega_{r}^{2}\right)}^{1/3}$ is the geometrical average of magnetic frequencies, and $a_{\mathrm{ho}}=\sqrt{\hbar /m\bar{\omega }}$ is the corresponding oscillator length. When suddenly released from the combined trap, the atomic gas undergoes a free expansion. After a time of flight of τ, the expanded subcondensates overlap completely with each other, and the wave function of the atomic cloud can be written as

$$\Psi \left(x,\tau \right)=\sum_{k=-k_{M}}^{k_{M}}\sqrt{N_{k}}\Phi \left(x-kd,\tau \right)e^{\frac{im}{2\hbar \tau }{\left(x-kd\right)}^{2}+i\phi_{k}^{r}},$$

where $\phi_{k}^{r}$ is a completely random phase which accounts for the loss of coherence between the subcondensates, while

$$\Phi \left(x-kd,\tau \right)=\pi^{-\frac{1}{4}}{\left(\sigma_{x}+i\hbar \tau /m\sigma_{x}\right)}^{-\frac{1}{2}}e^{-\frac{{\left(x-kd\right)}^{2}}{2{\left(\hbar \tau /m\sigma_{x}\right)}^{2}+2\sigma_{x}^{2}}}$$

refers to the expanding wavefunction initially localized to the Wannier function at the *k*th site [17,23]. The atomic density profile is then given by $n\left(x,\tau \right)={\left|\Psi \left(x,\tau \right)\right|}^{2}$.

In the numerical calculation of atomic densities, we used a high spatial resolution of 0.09μm, which was 100 times narrower than the pixel size. Then we convoluted the density profile with the point spread function of the imaging systems, which we approximated by a Gaussian function, $\exp\left(-x^{2}/2\sigma^{2}\right)/\sqrt{2\pi }\sigma$. In our case, the root mean square radius was about σ=4.5μm. The convoluted density profile was then binned in steps of 9 μm to account for the finite pixel size. Under the same conditions, we repeated the density calculation many times to obtain a set of density profiles, from which we extracted the density–density correlation function according to Equation (2).

**Figure 3:**
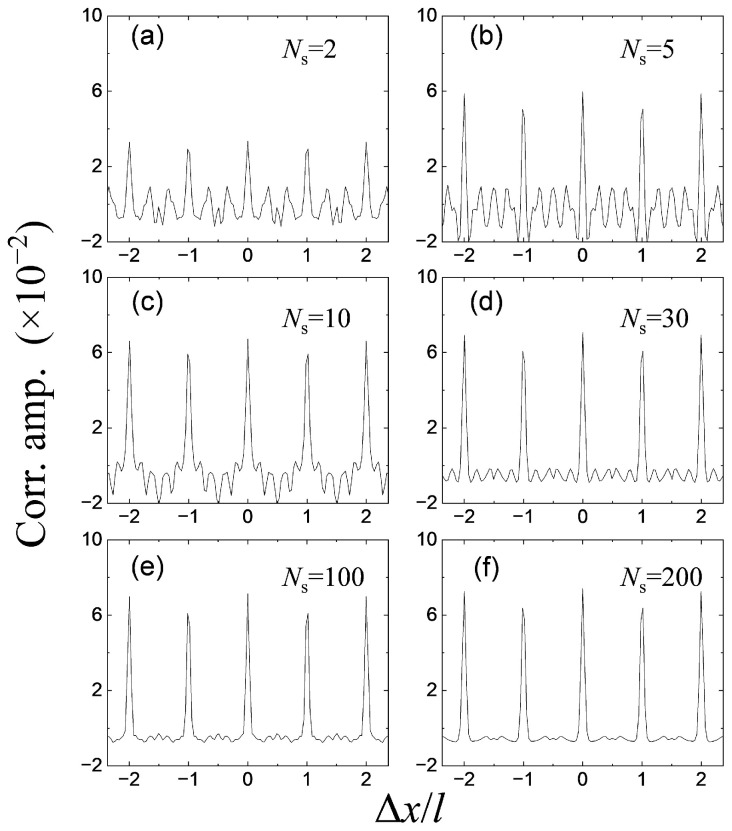
Density–density correlation spectra calculated from simulated density profiles. From (**a**–**f**), the number of density profiles used in calculation was $N_{s}=2,5,10,30,100$, and 200, respectively.

Figure 3 shows the density–density correlation spectra for different numbers of density profiles, $N_{s}$. For each density profile, $\phi_{k}^{r}$ is distributed uniformly between −π and π. As expected, each spectrum exhibited equally spaced peaks, with a spacing of $k_{L}$. Below $N_{s}=30$, the correlation signal increased with the increasing number of density profiles. However, the amplitude of each peak reached its maximum at $N_{s}∼30$, and remained unchanged beyond this point. In addition, the correlation peaks at the positions of ±2l were higher than those at the positions of ±l, which is consistent with our experimental observations. It is also clear that a larger number of density profiles is better for lower background fluctuations.

**Figure 4:**
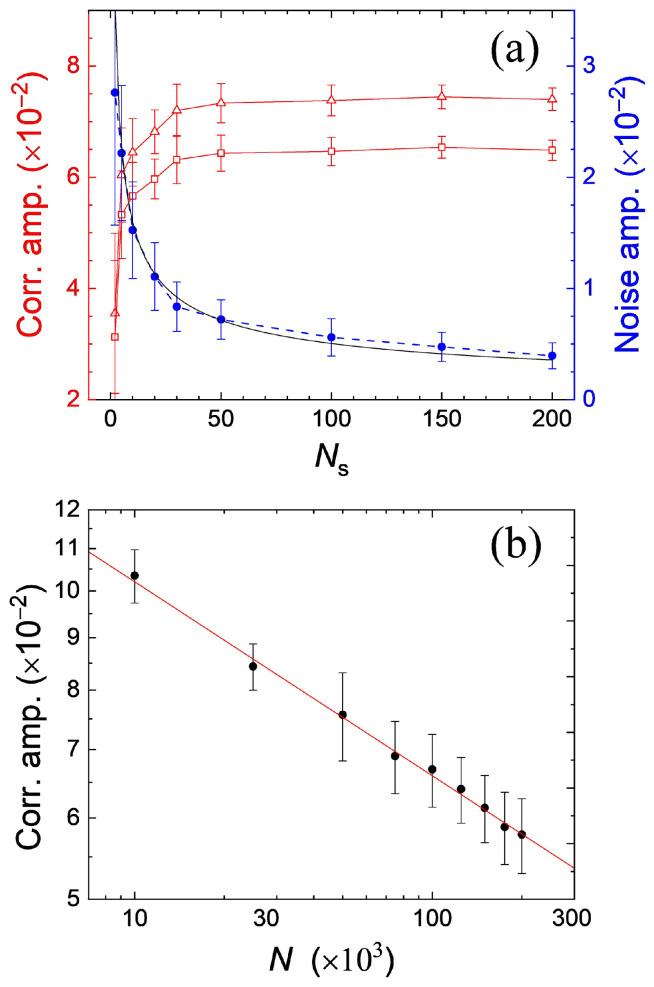
(**a**) The amplitude of correlation peaks and background noise level versus the number of density profiles repeatedly numerically generated. Open triangles and squares are the peak heights at the positions of ±2l and ±l, respectively. Solid dots are the peak-to-peak values of background fluctuation. The black line is a decaying curve in the form of $1/\sqrt{N_{s}}$. The red and blue lines are guides to the eye. (**b**) The amplitude of correlation peaks versus the number of atoms *N* loaded in the 1D lattice. The solid line is a fitted power-law decaying curve in the form of $N^{-\alpha }$. Each point corresponds to an average of 20 repeated calculations. The error bars denote root-mean-square deviations.

The dependence of the correlation peak heights and background fluctuations upon $N_{s}$ is displayed directly in Figure 4a. Each point represents an average of over 20 repeated calculations for a given $N_{s}$. The background fluctuation amplitude decreased with $N_{s}$, with a scaling of $∼1/\sqrt{N_{s}}$ (see the blue solid dots and the fitted black line in Figure 4a). The signal-to-noise (SNR) can reach 19 if 30 density profiles are used in the calculation of density–density correlation. In our experiment, the SNR was only ∼4 with 30 absorption images used. The higher noise level in the experiment may be attributed to the imaging noise arising from optical interference effects from the coherent illumination light [24].

Equation (3) indicates that the number of occupied lattice sites *M* is proportional to $N^{1/5}$. Then, the peak height $C(\Delta x_{\mathrm{peak}})$ should scale as $N^{-D/5}$. The simulated correlation peak height versus the total atom number in the optical lattice is plotted in Figure 4b. The power-law fitting to the data points yielded an exponent of ∼0.19, which roughly agrees with the theoretical value (D/5=0.2). Note that the scaling law $C\left(\Delta x_{\mathrm{peak}}\right)\propto N^{-D/5}$ had already been verified for the MI in 3D optical lattices [2].

## 4. Discussion and Conclusions

For ultracold atoms loaded in a 1D lattice, the lattice site number occupied by the atoms significantly affects the interference patterns of the ultracold bosonic gases in MI states. In an early experimental work [25], a high-contrast matter wave interference was observed for a chain of 30 independent BECs with uncorrelated phases. In contrast, with more occupied lattice sites, a 1D lattice system in MI state showed vanished interference patterns [12]. It has been pointed out that overlapping a large number of independent BECs with random phases will reveal the vanished visibility of the interference pattern [26]. In our experiments, the atomic gas occupied hundreds of lattice sites, and the disappearance of the interference pattern for the deep lattice was thus reasonable.

Unlike 3D lattice systems, where the atoms are strongly confined in three dimensions and each lattice well has only 1∼3 atoms [27], a 1D lattice system features pancake-shaped wells, where each is usually occupied by hundreds of atoms [17]. The wave packet has a Thomas–Fermi profile in the radial direction, due to the repulsive on-site interactions. Thus, the wave packet size in the radial directions is much larger than the single-particle ground-state wave packet [11,28], and the on-site interaction energy *U* is hence significantly reduced. If a single-particle ground-state wave packet is used in the calculation of *U*, the Mott phase transition would occur at a critical lattice depth of s=77, lower than the critical value (s=96) where the Thomas–Fermi profile of the wave packet is adopted. At the depth level of s=77, the on-site interaction energy U/h≈1.8 Hz for the case of the Thomas–Fermi profile, whereas *U* is seven times larger (U/h≈12.2 Hz) for the case of single-particle ground-state wave packet.

Experiments on density–density correlation usually need to repeatedly take a large number of absorption images. For example, 43 and 60 images were used for 3D [2] and 2D [3] lattices, respectively. Our results show that, for a 1D lattice system, 30 absorption images are enough to achieve a reliable spectrum, due to the strong correlation signal compared to higher-dimensional lattices.

Finite temperature effects could be neglected in our experiment since the lattice was loaded with a near pure condensate at a temperature below 50 nK. Fölling et al. investigated density–density correlation for a thermal gas well above the critical temperature ($T_{c}$), and they did not observe correlation peaks, despite there being no observable population in the excited Bloch bands [2]. Recently, Hanbury Brown and Twiss bunching was observed for the noncondensed fraction of an interacting Bose gas [29]. By loading a normal rubidium gas into the 1D lattice, we checked in an experiment the density–density correlation spectrum. No periodic narrow correlation peaks at positions of Δx/l=±1,2 were found for the normal gas at a temperature of $T=1.4T_{c}$, and only a central peak at Δx/l=0 existed.

In summary, we experimentally studied the normalized density–density correlation of the ultracold bosonic gases released from a 1D optical lattice. Periodic correlation peaks were observed for the optical lattice system deep in the MI regime. Our work further confirms that density–density correlation is indeed a powerful tool for revealing the hidden order in strongly correlated phases. In addition, by assigning a completely random phase to each wave packet in a single lattice site, we simulated the atomic density profiles of the released bosonic gases along the lattice direction, and further calculated the corresponding density–density correlation spectra. Our simulation results agreed with the experimental observations.

## Data Availability

Data are contained within the article.
